# Release of Active Peptidyl Arginine Deiminases by Neutrophils Can Explain Production of Extracellular Citrullinated Autoantigens in Rheumatoid Arthritis Synovial Fluid

**DOI:** 10.1002/art.39313

**Published:** 2015-11-25

**Authors:** Julia Spengler, Božo Lugonja, A. Jimmy Ytterberg, Roman A. Zubarev, Andrew J. Creese, Mark J. Pearson, Melissa M. Grant, Michael Milward, Karin Lundberg, Christopher D. Buckley, Andrew Filer, Karim Raza, Paul R. Cooper, Iain L. Chapple, Dagmar Scheel‐Toellner

**Affiliations:** ^1^ Arthritis Research UK Centre of Excellence for Rheumatoid Arthritis Pathogenesis and University of BirminghamBirminghamUK; ^2^ Karolinska University Hospital and Karolinska InstitutetStockholmSweden; ^3^ Karolinska InstitutetStockholmSweden; ^4^ University of BirminghamBirminghamUK; ^5^ Arthritis Research UK Centre of Excellence for Rheumatoid Arthritis Pathogenesis, University of Birmingham, and Sandwell and West Birmingham Hospitals NHS TrustBirminghamUK; ^6^ Arthritis Research UK Centre of Excellence for Rheumatoid Arthritis Pathogenesis, University of Birmingham, and University Hospitals NHS Foundation TrustBirminghamUK

## Abstract

**Objective:**

In the majority of patients with rheumatoid arthritis (RA), antibodies specifically recognize citrullinated autoantigens that are generated by peptidylarginine deiminases (PADs). Neutrophils express high levels of PAD and accumulate in the synovial fluid (SF) of RA patients during disease flares. This study was undertaken to test the hypothesis that neutrophil cell death, induced by either NETosis (extrusion of genomic DNA–protein complexes known as neutrophil extracellular traps [NETs]) or necrosis, can contribute to production of autoantigens in the inflamed joint.

**Methods:**

Extracellular DNA was quantified in the SF of patients with RA, patients with osteoarthritis (OA), and patients with psoriatic arthritis (PsA). Release of PAD from neutrophils was investigated by Western blotting, mass spectrometry, immunofluorescence staining, and PAD activity assays. PAD2 and PAD4 protein expression, as well as PAD enzymatic activity, were assessed in the SF of patients with RA and those with OA.

**Results:**

Extracellular DNA was detected at significantly higher levels in RA SF than in OA SF (*P* < 0.001) or PsA SF (*P* < 0.05), and its expression levels correlated with neutrophil concentrations and PAD activity in RA SF. Necrotic neutrophils released less soluble extracellular DNA compared to NETotic cells in vitro (*P* < 0.05). Higher PAD activity was detected in RA SF than in OA SF (*P* < 0.05). The citrullinated proteins PAD2 and PAD4 were found attached to NETs and also freely diffused in the supernatant. PAD enzymatic activity was detected in supernatants of neutrophils undergoing either NETosis or necrosis.

**Conclusion:**

Release of active PAD isoforms into the SF by neutrophil cell death is a plausible explanation for the generation of extracellular autoantigens in RA.

The synovial cavity of patients with rheumatoid arthritis (RA) is infiltrated by large numbers of neutrophils. Once activated, neutrophils augment disease development and progression through the generation of reactive oxygen species, proteolytic enzymes, and proinflammatory cytokines 1. Several forms of neutrophil cell death have been described 2, such as apoptosis, necrosis, and a novel pathway involving extrusion of genomic DNA–protein complexes known as neutrophil extracellular traps (NETs), termed NETosis 3. This complex process involves the active release of genomic DNA into the extracellular space. The resulting network of DNA and associated proteins (e.g., histones, myeloperoxidase, and neutrophil elastase), or NETs, is present in the joints of RA patients 4, 5. NETosis involves activation of peptidylarginine deiminase 4 (PAD4). The importance of this enzyme to NETosis is underlined by the observation that neutrophils in PAD4‐deficient mice do not generate NETs upon stimulation 6.

PAD4 belongs to a family of 5 PAD isoforms whose enzymatic activity leads to deimination of arginine side chains, resulting in protein citrullination. PAD isoforms are highly homologous and functionally similar, but differ in their expression pattern throughout various organ systems and cell types 7. Neutrophils express PAD2, which is ubiquitously expressed, and PAD4, which is mainly expressed by myeloid cells 7. Activation of PAD isoforms is relevant to RA, as specific autoimmunity to citrullinated proteins has been observed in ∼60% of RA patients 8, 9, and this characteristic defines a group of patients with more aggressive disease and distinct genetic associations.

Anti–citrullinated protein antibodies (ACPAs) bind a diverse range of citrullinated proteins, and B cell responses to these modified proteins have been observed in the rheumatoid joint 10. It is not yet clear whether neutrophils in the synovial fluid (SF) contribute to the pool of available extracellular PAD and whether PADs are enzymatically active. In this context, it is also important to know whether PAD isoforms are released attached to the DNA–protein complex of the NETs or whether they are freely diffusible. Neutrophils are rarely observed in synovial tissue, whereas they are the most abundant cell type in the SF 11. If PADs remain tethered to NETs, this would potentially limit the role of neutrophil‐derived PAD in the SF. However, the lack of a basement membrane or tight junctions in the synovial lining suggests that soluble, freely diffusible PAD released from neutrophils within the SF could enter synovial tissue and potentially contribute to the local production of autoantigens throughout the inflamed synovium 12.

We therefore investigated the release of PAD and citrullinated proteins from neutrophils undergoing NETosis and compared this process to necrosis. Moreover, the localization of PAD within the cells, the levels of PAD activity in SF from patients with different arthritides, and the levels of PAD activity in relation to evidence of neutrophil infiltration and NETosis were examined.

## PATIENTS AND METHODS

### Patient selection and sample collection

RA patients fulfilled the American College of Rheumatology 1987 classification criteria for RA 13. Osteoarthritis (OA) of the knee and psoriatic arthritis (PsA) were diagnosed according to established criteria 14, 15. SF samples were aspirated from the joints of all patients under manual palpation or ultrasound guidance. Synovial tissue was obtained by ultrasound‐guided synovial biopsy 16. Ethics approval was obtained and all participants gave their written informed consent. Details on the demographic and clinical characteristics of the patients are shown in Table 1.

**Table 1:** Demographic and clinical characteristics of the study patients^a^

	Patients with RA	Patients with PsA	Patients with OA
No. of patients	54	12	20
No. (%) female	36 (67)	4 (33)	13 (65)
Age, median (IQR) years	58 (46–66)	44 (40–50)	63 (52–67)
Disease duration, median (IQR) years	4 (1–9)	9 (5–19)	–
No. (%) RF positive	41 (76)	0 (0)	–
No. (%) ACPA positive^b^	35 (73)	0 (0)	–
CRP, median (IQR) mg/ml	19 (8–56)	9 (3–10)	–
ESR, median (IQR) mm/hour	30 (18–48)	6 (3–9)	–
SJC (of 28 joints), median (IQR)	5 (2–7)	1.5 (1–2)	–
TJC (of 28 joints), median (IQR)	5 (3–10)	2 (1–4)	–
DAS28‐ESR, median (IQR)	5 (4–6)	3 (2–3)	–
No. (%) receiving DMARDs	40 (74)	9 (75)	–

a Not all patient samples were used for the same experiments. For each assay, the maximum number of patient samples that were available at a given time point were processed. The number of patients from whom biologic material was obtained for each experiment is indicated throughout the text. RA = rheumatoid arthritis; PsA = psoriatic arthritis; OA = osteoarthritis; IQR = interquartile range; RF = rheumatoid factor; CRP = C‐reactive protein; ESR = erythrocyte sedimentation rate; SJC = swollen joint count; TJC = tender joint count; DAS28‐ESR = Disease Activity Score in 28 joints using the ESR; DMARDs = disease‐modifying antirheumatic drugs.

b Positivity for anti–citrullinated protein antibodies (ACPAs) was determined using an anti–cyclic citrullinated peptide test in samples available from 48 of 54 patients.

### Reagents

For immunofluorescence staining, we used antibodies to neutrophil elastase (ab21595; Abcam), combined with an Alexa Fluor 647–conjugated goat anti‐rabbit IgG antibody (Jackson ImmunoResearch), and antibodies to PAD4 (ab128086; Abcam), combined with a streptavidin–Alexa Fluor 488–conjugated (Jackson ImmunoResearch) goat anti‐mouse IgG2a biotin antibody (Southern Biotech). Anti‐human PAD2 antibodies (ab50257; Abcam) were revealed with an Alexa Fluor 488–conjugated donkey anti‐rabbit IgG antibody (Life Technologies). Antibodies to CD15 (clone MEM‐158; Immunotools) or to citrullinated histone H3 (ab5103; Abcam) were combined with a donkey anti‐mouse IgM antibody (Jackson ImmunoResearch) or an Alexa Fluor 647–conjugated donkey anti‐rabbit IgG antibody (Jackson ImmunoResearch), respectively. All stainings included concentration‐, species‐, and isotype‐matched control antibodies.

For Western blotting, antibodies specific for neutrophil elastase (ab21595; Abcam), antibodies specific for PAD2 (ab50257; Abcam), and 2 monoclonal antibodies to PAD4 (H00023569‐M01 from Novus Biologicals and ab128086 from Abcam) were used. All 3 PAD isoform antibodies were tested to exclude cross‐reactivity. Human recombinant PAD4 was purchased from Cayman Chemical (item no. 10500). Sytox Green was purchased from Invitrogen. Other reagents used included DNase I (AM2235; Ambion, Applied Biosystems), placental DNA (D4642; Sigma‐Aldrich), and protease inhibitor cocktail (P8340; Sigma‐Aldrich).

### Neutrophil isolation from peripheral blood

Peripheral blood from healthy donors was anticoagulated with EDTA (E7889; Sigma‐Aldrich) at a final concentration of 1.5 m*M*. Neutrophils were separated from the peripheral blood using Percoll discontinuous density gradients, as previously described 17.

### Immunofluorescence microscopy

Slide preparations of SF samples from all patients were created by pipetting 30 μl SF onto a glass slide. Cells were allowed to sediment for 2 minutes. Supernatant was carefully removed and slides were left to air dry and frozen at −20°C. SF slide preparations and 5‐μm frozen sections from RA synovial tissue were fixed in 4% paraformaldehyde (PFA) for 10 minutes, blocked with phosphate buffered saline (PBS) with 10% fetal calf serum (FCS), and incubated with primary antibodies to CD15 or to neutrophil elastase, diluted in 10% FCS in PBS. Nuclei were counterstained with Hoechst S769121 (Life Technologies).

For immunofluorescence staining of stimulated cells in vitro, neutrophils were seeded on coverslips and fixed with 4% PFA for 10 minutes after stimulation, and kept overnight at 4°C. Coverslips were rinsed with PBS and then blocked for 45 minutes with PBS in 2% bovine serum albumin (BSA), 2% goat and/or donkey serum, and 0.25% Triton X‐100 3. Primary antibodies were diluted in PBS with 2% BSA and 0.25% Triton X‐100, washed in PBS, revealed using Alexa Fluor– conjugated secondary antibodies, and counterstained with 10 μg/ml Hoechst S769121. Images were captured and processed with a Zeiss confocal LSM 510 laser scanning microscope.

### Isolation and quantification of NETs released during in vitro NETosis, for analysis by Western blotting and mass spectrometry

Neutrophils isolated from the peripheral blood of healthy donors were seeded at a concentration of 1 × 10^6^/ml and stimulated using phorbol myristate acetate (PMA) at a final concentration of 25 n*M* for 4 hours at 37°C in an atmosphere of 5% CO_2_. Supernatants of either stimulated or unstimulated neutrophils were harvested, and the wells were washed 3 times to remove unbound proteins, as described previously 18. Cell‐associated NETs were solubilized with 10 units/ml DNase I for 20 minutes in 500 μl RPMI medium and protease inhibitor cocktail at a dilution of 1:200 (P8340; Sigma‐Aldrich). The reaction was stopped with 5 m*M* EDTA. Supernatants of stimulated or unstimulated neutrophils from all 3 washing steps, as well as supernatants of the DNase I–treated NET fraction and the untreated control fraction, were collected. All supernatants were centrifuged for 10 minutes at 300*g* to remove whole cells, and for 10 minutes at 16,000*g* to remove debris. Proteins were precipitated with a final concentration of 15% weight/volume trichloroacetic acid for 20 minutes on ice, and then centrifuged for 10 minutes at 16,000*g*. Pellets were washed twice with ice‐cold acetone.

To quantify DNA, 90 μl of each supernatant was incubated with 10 μl of 10 μ*M* Sytox Green (Invitrogen) at a final concentration of 1 μ*M* in a black 96‐well assay plate (Corning) and incubated for 10 minutes at room temperature. Fluorescence intensity readings for DNA quantification were obtained with a plate reader (BioTek‐Synergy 2) at a wavelength of 530 nm, and the values were compared to a standard curve of values for purified human DNA (Sigma‐Aldrich).

### Induction of necrotic cell death in neutrophils.

Neutrophils were placed in 2‐ml polypropylene tubes at a cell concentration of 1 × 10^6^/ml in 1 ml and subjected to 5 freeze–thaw cycles, as described previously 19. After the last freeze–thaw cycle, tubes were centrifuged for 10 minutes at 300*g* to remove whole cells, and the supernatants were transferred to fresh tubes and centrifuged a second time for 10 minutes at 16,000*g* to remove debris. PAD activity and DNA concentrations were assessed in supernatants of resting (unstimulated) neutrophils, necrotic neutrophils, and neutrophils stimulated with 25 n*M* PMA to induce NETosis (all derived from peripheral blood samples from the same healthy donors).

### Western blotting

Protein precipitates were solubilized in sodium dodecyl sulfate–polyacrylamide gel electrophoresis (SDS‐PAGE) sample buffer, separated on 12% SDS‐PAGE gels, and transferred to PVDF membranes using a wet blotting system. Human skeletal muscle tissue was lysed in radioimmunoprecipitation assay buffer (R0278; Sigma‐Aldrich) in combination with a protease inhibitor cocktail (P8340; Sigma‐Aldrich) and diluted with SDS‐PAGE sample buffer after protein quantification. After blocking with 5% milk powder in Tris buffered saline (TBS) with 0.05% Tween 20, membranes were incubated overnight at 4°C with primary antibody and developed with horseradish peroxidase (HRP)–conjugated antibody and ECL Plus or ECL Prime reagents (GE Healthcare, Amersham).

### Quantification of extracellular DNA in SF samples using Sytox Green

Untreated SF samples used immediately after aspiration from the joints were diluted with PBS to 1:10, 1:100, and 1:1,000, and levels of free DNA were assessed in the SF in same manner as described above for DNA quantification in supernatants, with the fluorescence intensity readings performed at a wavelength of 530 nm. DNA concentrations were calculated with reference to a standard curve of values for purified DNA (Sigma‐Aldrich). To obtain cell‐free SF, the samples were centrifuged for 10 minutes at 300*g*.

### Quantification of neutrophils and macrophages in SF samples

SF samples were stained with Giemsa (Diff‐Quick; Gamidor Technical Services). Thereafter, total cell counts and the proportion of neutrophils and macrophages within the synovial infiltrate were determined using a hemocytometer and cytospin preparations.

### Mass spectrometry analysis

Supernatants and NET fractions from stimulated neutrophils from 7 healthy donors were digested by trypsin 20 and analyzed by LTQ OrbitrapVelos ETD or by Q Exactive mass spectrometry (Thermo Fisher Scientific). Data were quantified using Quanti work flow 21. *P* values were calculated using Student's *t*‐test, and expectation values were calculated by multiplying the *P* values by the number of observations (for further details, see Supplementary Methods, available on the *Arthritis & Rheumatology* web site at http://onlinelibrary.wiley.com/doi/10.1002/art.39313/abstract).

### Depletion of albumin from the SF

SF samples were centrifuged at 300*g*. Albumin was removed from the supernatants using a Maxi ProteoExtract Albumin/IgG Removal Kit (Calbiochem). The protein concentration in the eluate was determined using a BCA Protein Assay Kit (Thermo Fisher Scientific). Twelve micrograms of each sample was diluted with 5× SDS loading buffer and further used for SDS‐PAGE and Western blotting analyses.

### Detection of citrulline modifications

Citrullinated residues in proteins from neutrophil supernatants blotted on PVDF membranes were modified using the protocol described by Senshu et al 22. Briefly, membranes were incubated overnight at 37°C with an end concentration of 0.77 m*M* FeCl_3_, 1.47*M* H_3_PO_4_, and 2.25*M* H_2_SO_4_ in combination with 0.25*M* C_2_H_4_O_2_, 25 m*M* 2,3‐butanedione monoxime, and 6.64 m*M* antipyrine. After rinsing with deionized H_2_O and blocking with 5% milk powder in TBS with 0.05% Tween 20 the next day, membranes were probed overnight at 4°C with a monoclonal human anti–modified citrulline antibody (clone C4; Modiquest) and developed with an HRP‐conjugated goat anti‐human antibody (Jackson ImmunoResearch) and ECL Prime (GE Healthcare, Amersham).

### PAD activity assay

For analysis of PAD activity released by stimulated neutrophils in vitro, isolated cells were seeded at 1 × 10^6^/ml and left to sediment for 1 hour at 37°C in an atmosphere of 5% CO_2_ in RPMI 1640 medium (Sigma‐Aldrich). After sedimentation, the supernatant was replaced either with 1 ml prewarmed RPMI medium (unstimulated control) or with 1 ml of 25 n*M* PMA in RPMI medium. After stimulation, the supernatants were collected and treated with protease inhibitor cocktail (diluted 1:100; Sigma‐Aldrich) with an additional 3.5 m*M* 4‐(2‐aminoethyl)benzenesulfonyl fluoride hydrochloride on ice. Supernatants were centrifuged at 4°C for 5 minutes at 300*g* to remove residual cells and diluted 1:2 with deimination buffer (8.58 m*M* CaCl_2_, 5 m*M* dithiothreitol [DTT], and 40 m*M* Tris HCl, pH 7.5), which, together with the 0.42 m*M* calcium in RPMI 1640 medium, resulted in a calcium concentration of 4.5 m*M*. Supernatants were transferred to an Antibody Based Assay for PAD activity (ABAP) (MQ‐17.101‐96; ModiQuest Research), incubated for 1.5 hours at 37°C, and further processed according to the manufacturer's protocol.

RA SF and OA SF were centrifuged to remove cells, and the supernatants were cryopreserved. To compare PAD activity at native and artificial calcium concentrations, 100 μl SF was either loaded into the ABAP assay directly or prediluted 1:100 with deimination buffer (5 m*M* CaCl_2_, 1 m*M* DTT, and 40 m*M* Tris HCl, pH 7.5).

### Statistical analysis

Data were analyzed using GraphPad Prism software (version 5.0). The individual statistical tests used were the Mann‐Whitney U test, Wilcoxon's matched pairs signed rank test, and Spearman's test for correlations. *P* values less than 0.05 were considered significant.

## RESULTS

### Presence of free extracellular DNA in RA SF

**Figure 1:**
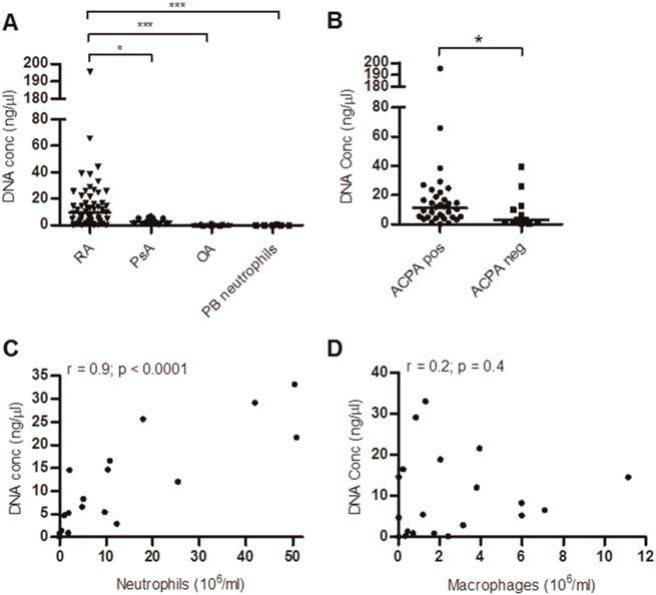
Presence of extracellular DNA in the synovial fluid (SF) of patients with rheumatoid arthritis (RA) and correlation with neutrophil cell counts. **A,** Quantification of extracellular DNA concentrations (conc) in the SF of patients with RA (n = 54), patients with psoriatic arthritis (PsA) (n = 12), and patients with osteoarthritis (OA) (n = 15). Freshly isolated neutrophils from the peripheral blood (PB) of healthy donors (n = 6) were used as a negative control. **B,** Comparison of free DNA levels in the SF between anti–citrullinated protein antibody (ACPA)–positive and ACPA‐negative RA patients. In **A** and **B,** symbols represent individual patients; horizontal bars show the median. **∗** = *P* < 0.05; **∗∗** = *P* < 0.01; **∗∗∗** = *P* < 0.001, by Mann‐Whitney U test. **C** and **D,** Assessment of correlations between DNA levels and the neutrophil concentration **(C)** and macrophage concentration **(D)** in the SF of 20 RA patients. Correlations were determined by Spearman's rho test.

Quantification of extracellular DNA in freshly obtained, not cryopreserved, SF samples from patients with RA, patients with OA, and patients with PsA confirmed and extended previous findings indicating that free extracellular DNA can be found in RA SF. Samples were compared to a known DNA standard solution in individual experiments. Significantly higher DNA levels were found in SF from RA patients compared to SF from patients with OA (*P* < 0.001) and patients with PsA (*P* < 0.05) (Figure 1A). Furthermore, significantly higher levels of extracellular DNA were detected in SF from ACPA‐positive RA patients compared to ACPA‐negative RA patients (*P* < 0.05) (Figure 1B). A small number of patients with different types of inflammatory arthritis, such as gout (n = 2), pseudogout (n = 1), or reactive arthritis (n = 1), showed variable levels of extracellular DNA in the SF (data not shown).

Validation experiments in which the DNA concentration in untreated SF samples (n = 12) was measured before and after centrifugation of the cells showed that 78% of the signal (interquartile range 66–89%) was derived from cell‐free SF samples, suggesting that necrotic cells with permeable membranes contributed only a small proportion of the DNA signal (see Supplementary Figure 1A, available on the *Arthritis & Rheumatology* web site at http://onlinelibrary.wiley.com/doi/10.1002/art.39313/abstract). In parallel experiments, the release of free extracellular DNA was compared between neutrophils undergoing NETosis and neutrophils undergoing necrosis. As shown in Supplementary Figure 1B (available on the *Arthritis & Rheumatology* web site at http://onlinelibrary.wiley.com/doi/10.1002/art.39313/abstract), a significantly higher amount of soluble extracellular DNA was released from neutrophils after NETosis as compared to that in unstimulated cells and that in necrotic cells (both *P* < 0.05).

Levels of free DNA in SF samples showed a strong correlation with neutrophil cell counts in the SF of RA patients (Figure 1C). In contrast, no correlation of free DNA levels with macrophage counts in the RA SF could be observed (Figure 1D). Moreover, no statistically significant correlations between DNA levels in the RA SF and clinical parameters, such as the Disease Activity Score in 28 joints, the C‐reactive protein level, or the erythrocyte sedimentation rate, were observed.

**Figure 2:**
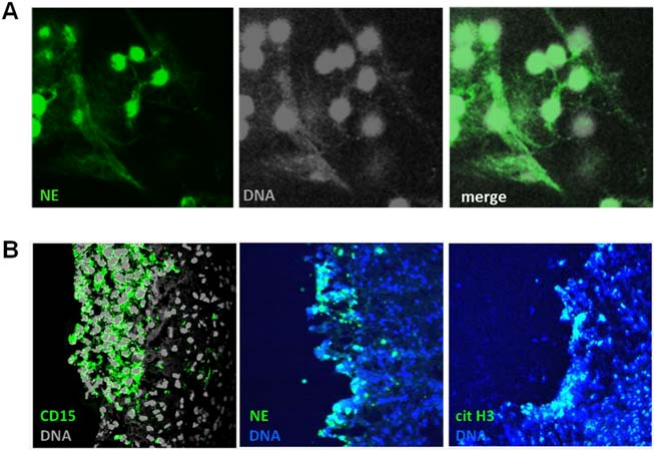
Detection of neutrophil extracellular traps in the synovial fluid (SF) of patients with rheumatoid arthritis (RA) and attached to the synovial tissue of RA patients. **A,** SF preparations from 17 RA patients were stained for extracellular DNA (gray) and neutrophil elastase (NE) (green). **B,** Neutrophil aggregates attached to the synovial tissue of 3 RA patients were detected by confocal laser scanning microscopy. Samples were labeled with antibodies specific for CD15, NE, and citrullinated histone 3 (cit H3) (green) as well as for DNA (gray and blue). Original magnification × 630.

Immunostaining of preparations of RA SF on glass slides showed a network of extracellular DNA strands, similar to that reported previously as a typical feature of NETs in a range of studies. The findings from immunostaining indicated that the extracellular DNA colocalized with neutrophil elastase, a major constituent of NETs, in the RA SF (Figure 2A). In frozen sections of synovial tissue from patients with RA, neutrophil aggregates were observed on the surface of the synovial lining, facing the joint cavity. As shown in Figure 2B, these aggregates stained positive for CD15 and neutrophil elastase. Extracellular DNA and citrullinated histone H3, a hallmark of NETosis, were observed to be associated with these aggregates (Figure 2B). These results are consistent with the concept that extracellular DNA and NETs are associated with the localization and number of neutrophils present in the SF of patients with inflammatory arthritis.

### In vitro PAD release during NETosis

**Figure 3:**
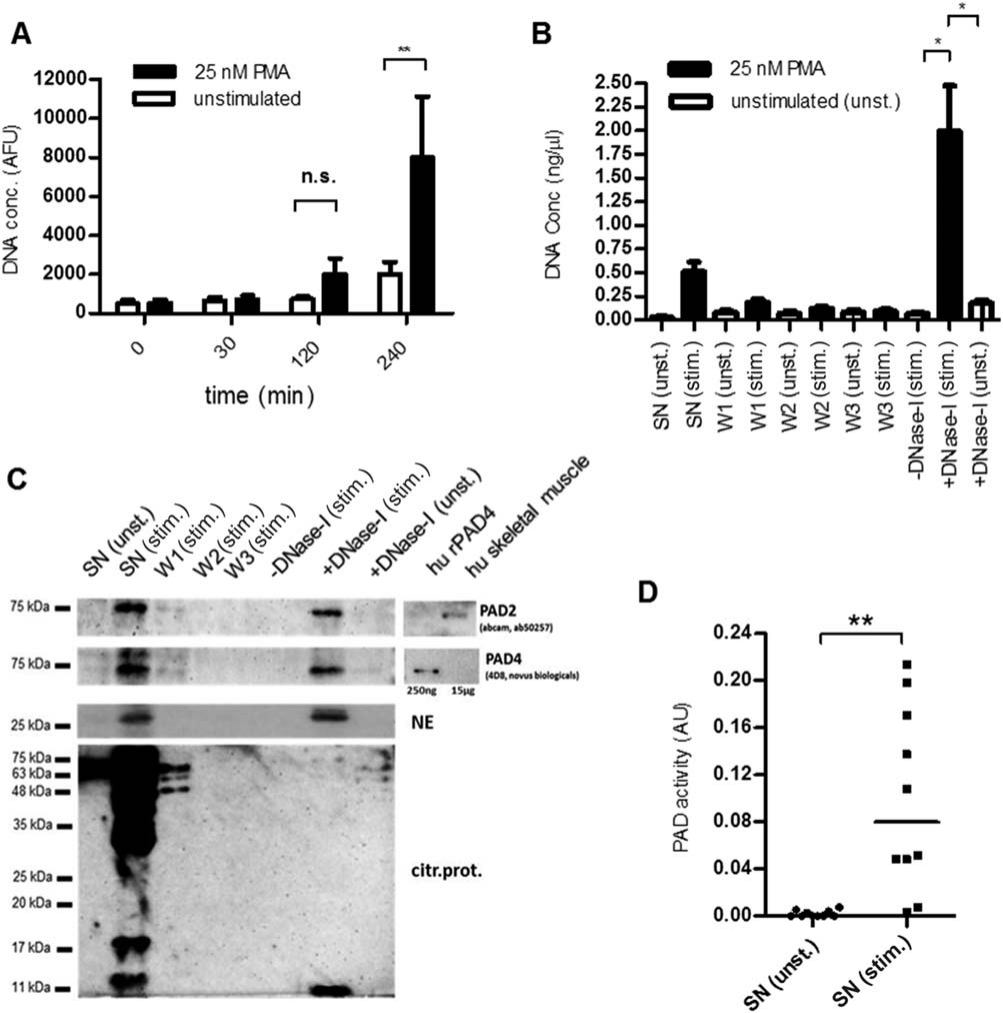
Release of peptidyl arginine deiminases (PADs) into the supernatant during NETosis, and detection of enzymatically active PADs. **A,** DNA release in peripheral blood neutrophils from healthy donors that were left unstimulated or stimulated with 25 m*M* phorbol myristate acetate (PMA) was assessed for up to 240 minutes after stimulation. Bars show the mean ± SD of 8 samples per group. **B,** For isolation of neutrophil extracellular traps, supernatants (SN) of unstimulated (unst.) or stimulated (stim.) neutrophils were collected. Subsequently, the cells were washed 3 times with RPMI medium (W1–W3), and stimulated cells were incubated in the absence or presence of DNase I; unstimulated cells treated with DNase I served as a control. Bars show the mean ± SD DNA concentration (conc) in 7 samples per group. **C,** Proteins (PAD2, PAD4, and neutrophil elastase [NE]) were precipitated from the same supernatants of unstimulated or stimulated cells as described in **B** and analyzed by Western blotting. Citrullinated proteins (citr.prot.) were detected using chemical modification and anti–modified citrulline antibody. PAD2 and PAD4 antibodies were tested for cross‐reactivity using human (hu) recombinant PAD4 (rPAD4) (250 ng) and human skeletal muscle tissue lysate (15 μg). One representative blot of 4 independent experiments is shown for each group. **D,** PAD activity in supernatants of unstimulated and stimulated cells was compared. Symbols represent individual donors (n = 10); bars show the median. **∗** = *P* < 0.05; **∗∗** = *P* < 0.01, by Wilcoxon's matched pairs signed rank test. AFU = arbitrary fluorescence units; NS = not significant.

Several of the proteins (e.g., fibrinogen and collagen) that can be converted into autoantigens by citrullination are predominantly located in the extracellular space. To understand the production of these citrullinated neoantigens, it is important to investigate sources of extracellular PAD. Since NETosis involves release of cellular components, we investigated whether neutrophils undergoing NETosis would release PAD into the extracellular space. In order to assess the release of unbound PAD into the supernatant and its attachment to NETs, an in vitro assay of NET isolation and detection was developed based on a previously published method 18. To validate the assay, DNA release during NETosis was assessed over a time course of up to 240 minutes (Figure 3A), and DNA levels in the fractions generated during NET isolation were tracked (Figure 3B). As shown in Figure 3B, only a small proportion of the DNA was detectable in the supernatant of stimulated neutrophils, suggesting that NETs are largely still attached to the neutrophil layer at this stage.

The stimulation was followed by 3 washing steps to ensure that only proteins attached to NETs were isolated. DNase I treatment released a fraction containing significantly higher levels of DNA compared to the fraction derived from DNase I–treated unstimulated neutrophils or the fraction derived from stimulated neutrophils incubated without DNase I (Figure 3B).

Both PAD2 and PAD4 were detected in the supernatant of stimulated neutrophils and in the NET fraction, suggesting that PAD2 and PAD4 are both released as freely diffusible proteins as well as bound to NETs (Figure 3C). Importantly, the antibodies used to detect PAD2 and PAD4 did not show cross‐reactivity (Figure 3C).

To validate our results, a quantitative proteomics analysis was performed. Consistent with the findings from a study by Urban et al 18, only a small number of proteins, comprising mainly granular and nuclear proteins, could be confirmed to be present in NETs, as compared to the wide range of proteins released into the supernatant during NETosis (see Supplementary Table 1, available on the *Arthritis & Rheumatology* web site at http://onlinelibrary.wiley.com/doi/10.1002/art.39313/abstract). Consistent with the findings from Western blot analysis, PAD2 and PAD4 were detected in both the supernatant and the DNase I–treated NET fraction of neutrophils from 7 patients. PAD2 was more abundant in the supernatant, whereas PAD4 was more abundant in the NET fraction (see Supplementary Table 1).

To test whether the activation of PAD isoforms during NETosis would result in the generation of citrullinated proteins, proteins from the culture supernatants were modified according to the protocol described by Senshu et al 22 and detected using an anti–modified citrulline antibody. As shown in Figure 3C, a large number of citrullinated proteins were released from stimulated neutrophils into the supernatant after 4 hours, compared to only a small number of citrullinated proteins present in the DNase I–treated NET fraction of stimulated neutrophils. Consistent with these findings, citrullinated peptides from several proteins could be identified by mass spectrometry (see Supplementary Table 2, available on the *Arthritis & Rheumatology* web site at http://onlinelibrary.wiley.com/doi/10.1002/art.39313/abstract).

Furthermore, analysis of PAD activity with a recently developed assay 23 showed higher levels of PAD activity in the supernatants of stimulated neutrophils after 2.5 hours of stimulation in vitro, compared to that in supernatants of unstimulated cells (Figure 3D). PAD activity was also observed in the supernatants of necrotic cells (see Supplementary Figure 1C, available on the *Arthritis & Rheumatology* web site at http://onlinelibrary.wiley.com/doi/10.1002/art.39313/abstract).

**Figure 4:**
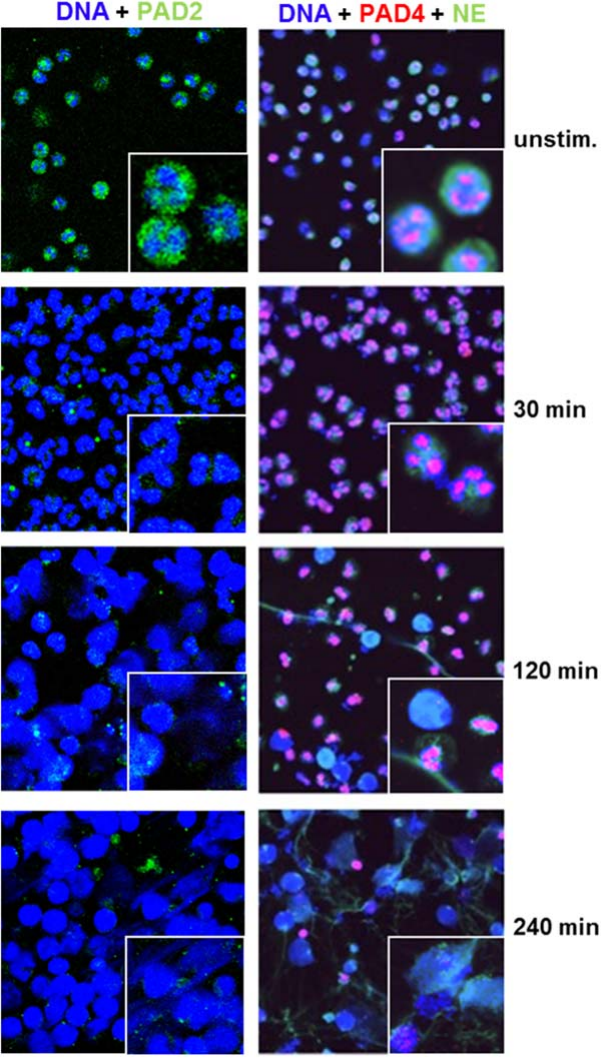
Time course of peptidyl arginine deiminase 2 **(**PAD2) and PAD4 release during NETosis. Unstimulated (unstim.) peripheral blood neutrophils from healthy donors and neutrophils at 30, 120, or 240 minutes after stimulation were assessed by confocal microscopy, revealing that a proportion of cells enter NETosis after 120 minutes of stimulation. In resting neutrophils, PAD2 is seen only in the cytosol, whereas PAD4 is localized in the nucleus. PAD2 levels drop within 30 minutes after stimulation, and PAD4 is lost from the nuclei of cells undergoing DNA decondensation. No PAD4 signal can be detected after 240 minutes of stimulation; these cells have reached a stage of NETosis at which the nuclear morphology has changed. DNA is shown in blue, PAD2 in green, PAD4 in red, and colabeling with neutrophil elastase (NE) in green. **Insets** show higher‐magnification views. Original magnification × 400; × 1,200 in **insets**.

Localization of PAD2 and PAD4 within the neutrophils undergoing NETosis was visualized by immunofluorescence staining (Figure 4). While PAD2 was largely restricted to the cytosol, PAD4 localization in resting peripheral blood neutrophils was found to be restricted to the cell nucleus, findings that were consistent with those in a study by Nakashima et al 24. Upon stimulation of the cells for 120 and 240 minutes, a proportion of the cells had changed their nuclear morphology. Nuclei were rounded and decondensed, indicating a stage of NETosis directly preceding DNA release (Figure 4). PAD2 staining was reduced within 30 minutes of stimulation, while PAD4 staining was lost from the nuclei, together with evidence of DNA decondensation. These results suggest that upon activation, neutrophils entering NETosis activate PAD isoforms, leading to citrullination of a large number of neutrophil proteins and also release of active PAD into the extracellular space.

### Evidence of enzymatically active PAD in RA SF

**Figure 5:**
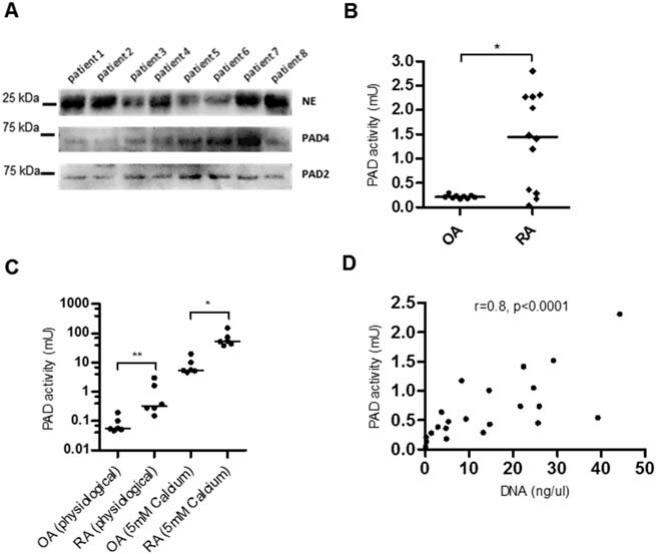
Detection of enzymatically active peptidyl arginine deiminase (PAD) in the synovial fluid (SF) of patients with rheumatoid arthritis (RA). **A,** Immunoblotting of albumin‐depleted SF samples from 8 RA patients shows presence of neutrophil elastase (NE), PAD4, and PAD2. **B,** PAD activity in the SF of patients with RA (n = 12) was significantly higher than that in the SF of patients with osteoarthritis (OA) (n = 9). **C,** PAD activity was observed in the OA SF and RA SF both at physiologic calcium concentrations and at higher calcium concentrations (5 m*M*). In **B** and **C,** symbols represent individual patients; horizontal bars show the median. **D,** PAD activity is significantly correlated with DNA levels in the SF of RA patients (n = 22). Data were analyzed using Spearman's rho test for correlation. **∗** = *P* < 0.05; **∗∗** = *P* < 0.01, by Mann‐Whitney U test.

In accordance with the results from a study by Kinloch et al 25, PAD2 and PAD4 proteins were detected in the cell‐free SF of patients with RA, in addition to the presence of neutrophil elastase (Figure 5A). Whereas PAD4 varied in its expression level, the amount of PAD2 was found to be more similar between patients. Furthermore, PAD enzymatic activity was significantly higher in the SF of RA patients than in the SF of OA patients (*P* < 0.05) at the supraphysiologic calcium concentrations used in this assay (Figure 5B). Interestingly, this significant difference between RA and OA could also be observed in undiluted SF samples at their native calcium concentrations, with PAD activity in the OA SF samples being ∼250‐fold lower than in the RA SF samples (Figure 5C).

Furthermore, PAD activity in fresh, untreated SF samples from patients with RA strongly correlated with not only the levels of extracellular DNA (ρ = 0.8, *P* < 0.0001) (Figure 5D) but also the neutrophil counts (ρ = 0.8, *P* = 0.002) and total cell counts (ρ = 0.72, *P* = 0.002) (see Supplementary Figures 1D and E, available on the *Arthritis & Rheumatology* web site at http://onlinelibrary.wiley.com/doi/10.1002/art.39313/abstract).

## DISCUSSION

The generation of citrullinated autoantigens after protein deimination by PAD is a key stage in the autoimmune response in ACPA‐positive patients with RA 8. Nevertheless, the mechanisms behind the activation of PAD, as well as the sites and circumstances of citrullination of autoantigens in RA, have remained unclear.

In this study, NETosis was identified as a source of freely diffusible, enzymatically active PAD, as well as a source of citrullinated proteins, suggesting that neutrophils have a central role in the generation of autoantigens. Neutrophils are the most abundant cell type present in the SF of patients with RA 11, 26. Evidence from this and other studies shows that neutrophils undergo NETosis at the site of inflammation and thereby release decondensed DNA 5, 27, 28, 29.

Extracellular DNA was observed within neutrophil infiltrates attached to the surface of the synovial lining layer and in SF preparations. Our study cannot exclude the possibility of cells other than neutrophils contributing to the production of extracellular DNA and to PAD activity in the SF of RA patients. As described previously, monocytes and macrophages can be a source of PAD 30, and cells such as eosinophils, mast cells, or macrophages can release their chromatin in a process related to NETosis 31. However, eosinophils and mast cells are present in only very low numbers in RA SF 32. In the present study, extracellular DNA levels in the RA SF showed no significant correlation with macrophage counts, whereas there was a highly significant association between DNA levels and neutrophil numbers. Comparison of neutrophils undergoing necrosis and those undergoing NETosis showed that there is significantly less release of soluble extracellular DNA from necrotic neutrophils. This difference may be explained by the high level of nuclear decondensation during the process of NETosis.

Consistent with our finding of PAD activity in the SF, several studies have already described the presence of citrullinated proteins in the SF of patients with inflammatory arthritis 25, 33, 34. Interestingly, some of these targets, such as citrullinated myeloid nuclear differentiation antigen or vimentin, were also generated during in vitro NETosis in our assay, thus further supporting the idea that NETs are a possible source for these citrullinated proteins in the joints. This is also consistent with other published work in which citrullinated proteins within NETs were described as targets of the autoimmune response both in RA and in Felty's syndrome 35, 36.

The number of citrullinated proteins identified in the present study was fairly small. The very rigorous validation process used to minimize false‐positive results, as described in the Supplementary Methods and Tables, may have contributed to this low number. The view that NETosis contributes to citrullination was also supported by findings from a study by De Rycke et al, in which localization of citrullinated proteins within extrasynovial deposits of polymorphonuclear cells was observed on the surface of the lining layer in RA patients 37.

Our data indicate that extracellular DNA levels and neutrophil concentrations in the SF correlated with PAD activity, which further supports the proposition that PADs are released and activated as a result of NETosis in the joints of patients with RA, in contrast to that observed in the SF of patients with OA, which showed minimal PAD activity. Release of PAD activity was also observed under conditions of induced necrosis in vitro. This is likely to involve release of cytosolic PAD2 after loss of membrane integrity.

PAD enzymatic activity is regulated by calcium ions 38. Indeed, intracellular Ca^2+^ levels were below the level needed for in vitro PAD activity, indicating that further, as‐yet undefined, regulatory mechanisms may be operating in the joints. Thus, in vitro PAD activity was initially measured at supraphysiologic calcium concentrations. However, although the PAD activity measured in RA SF at physiologic calcium concentrations was lower, it was still detectable with the ABAP assay used in this study and was significantly higher than that in OA SF. This observation was also confirmed in a recent small study using human fibrinogen as the substrate for PAD activity 39. In accordance with our data, calcium concentrations in RA SF samples in that study were found to be still sufficient to support PAD activity 39. It can therefore be concluded that the conditions for optimal activity in vitro differ from the conditions in vivo.

In this regard, a recent study by Darrah et al demonstrated a mismatch between the in vitro and in vivo calcium requirements for PAD4 activity, and explained this by the presence of PAD3/PAD4 cross‐reactive antibodies that may have a major role in decreasing the enzyme's requirement for calcium concentrations into the physiologic range in vivo 40. Moreover, recombinant and native PAD may differ in folding, posttranslational modification, and specific activity, further complicating the interpretation of the calcium dependency across experiments. More relevant for the patient, however, is the observation that PAD activity is being generated under the conditions present in the inflamed joint. Future studies will be needed to identify the contribution of different isoenzymes to the overall PAD activity observed during NETosis and to investigate regulatory factors that might modulate the enzymatic activity.

Since the assay used for measuring PAD activity does not distinguish between different PAD isoforms, it is possible that there is a separate, unique contribution to total PAD activity by PAD2, PAD3, and PAD4. Interestingly, mass spectrometry did not identify any unique peptides matching PAD3 as being present in neutrophils. Identification of the PAD isoforms that are responsible for the observed PAD activity is of interest, because it was recently reported that different PAD enzymes display distinct substrate specificities 41 and could therefore lead to the citrullination of different autoantigens.

Several studies have recognized the absence of synovial inflammation (histologically and by imaging) in individuals who have RA‐specific autoantibodies and joint pain but no clinically apparent joint swelling 42, 43, 44, and these studies therefore propose the concept that the initiating event leading to ACPA production is more likely to be located outside the joint. In this context, Rosenstein et al first hypothesized that the generation of citrullinated antigens by PPAD, the bacterial PAD enzyme derived from *Porphyromonas gingivalis* in periodontal tissue, could be a potential trigger for the initiation of ACPAs 45. In addition, the lungs have also been suggested to play a role in triggering the autoimmune response to citrullinated proteins 46. Based on findings of the presence of NETs in inflamed gingival crevicular fluid 29 and lungs 28, it is therefore also possible that PAD release during NETosis contributes to generation of citrullinated proteins initiating the ACPA response at these sites. In addition, other mechanisms, such as the generation of major histocompability complex–citrullinated peptide complexes in autophagosomes of antigen‐presenting cells leading to the induction of autoimmunity, are possible 47.

We therefore do not propose that production of citrullinated proteins during NETosis in the joint represents the original breakdown of immune tolerance to citrullinated proteins. Rather, we suggest that release of PAD from neutrophils in the joints of RA patients represents a later event in the disease process, during which citrullinated proteins and preexisting ACPAs form proinflammatory immune complexes and drive a continuous inflammatory response in the joints, which may be supported by our finding of significantly higher DNA levels in ACPA‐positive RA patients compared to ACPA‐negative RA patients. In this context, findings of a recent study suggesting that PAD4 is not essential for disease in the murine K/BxN autoantibody‐mediated model of arthritis does not conflict with our observations, as this model does not depend on autoimmunity to citrullinated proteins 48.

NETosis may not be the only source of citrullinated proteins in the joints of patients with inflammatory arthritis. Intracellular citrullination was reported in several cell populations in the synovium 12, 49 and in SF cells 50, and release of enzymatically active PAD could also be observed in our study after the induction of necrosis. Cell lysis induced by immune‐mediated membranolytic pathways, for example, could represent another source of PAD and citrullinated proteins 50. Nevertheless, further studies are needed to examine the mechanisms underlying the initial intracellular activation of PAD in relation to different cell death pathways.

ACPAs are enriched in SF compared to serum 51, and B cell clones specific for citrullinated proteins persist in the inflamed synovium of RA patients 10. This local immune response requires continuous production of citrullinated antigens in the joint. We therefore propose that activated neutrophils undergoing NETosis in the joints of patients with RA contribute to this supply of autoantigens by the release of enzymatically active PAD isoforms.

## AUTHOR CONTRIBUTIONS

All authors were involved in drafting the article or revising it critically for important intellectual content, and all authors approved the final version to be published. Dr. Scheel‐Toellner had full access to all of the data in the study and takes responsibility for the integrity of the data and the accuracy of the data analysis.

### Study conception and design

Spengler, Milward, Lungberg, Buckley, Filer, Raza, Cooper, Chapple, Scheel‐Toellner.

### Acquisition of data

Spengler, Lugonja, Ytterberg, Creese, Pearson, Scheel‐Toellner.

### Analysis and interpretation of data

Spengler, Lugonja, Ytterberg, Zubarev, Grant, Lundberg, Raza, Scheel‐Toellner.

## Supporting information


**Supplementary Table 1.** Quantitative comparison of protein levels by mass spectrometry in supernatants of stimulated neutrophils (SN) and DNase I–treated NET fraction of stimulated neutrophils (+DNase‐I and/or D) after in vitro induced NETosis.
**Supplementary Table 2.** Citrullinated proteins detected by mass spectrometry in supernatants of stimulated neutrophils (SN) and NET fraction (D) after in vitro induced NETosis
**Supplementary Figure 1. A,** Comparison of extracellular DNA levels (ng/μl) in untreated and cell‐free (centrifuged) SF samples in 12 patients with inflammatory arthritis. **B,** Neutrophils from healthy donors (n = 6) were either frozen and thawed to induce necrosis, or stimulated with PMA to induce NETosis. Release of DNA is shown in **B** and release of PAD activity in **C**. Statistical analysis was performed using Wilcoxon matched‐pairs signed rank test with horizontal bars representing median values (n = 6; n.s. = not significant; * indicates *P* < 0.05). **D,** PAD activity correlated significantly with neutrophil cell counts in the synovial fluid of RA patients (n = 13). Data were analyzed using Spearman's test for correlation (rho = 0.8, *P* = 0.002). **E,** PAD activity correlated significantly with the total cell count in untreated synovial fluid of RA patients (n = 15). Data were analyzed using Spearman's test for correlation (rho = 0.72, *P* = 0.002).
