# Supplementary material for: Release of Active Peptidyl Arginine Deiminases by Neutrophils Can Explain Production of Extracellular Citrullinated Autoantigens in Rheumatoid Arthritis Synovial Fluid
Source: Arthritis Rheumatol. 2015 Nov 25;67(12):3135–45. doi: 10.1002/art.39313 (PMC4832324; doi:10.1002/art.39313)
Supplement: Supplementary file 1 — Supplementary Table 1. Quantitative comparison of protein levels by mass spectrometry in supernatants of stimulated neutrophils (SN) and DNase I–treated NET fraction of stimulated neutrophils (+DNase‐I and/or D) after in vitro induced NETosis. Supplementary Table 2. Citrullinated proteins detected by mass spectrometry in supernatants of stimulated neutrophils (SN) and NET fraction (D) after in vitro induced NETosis Supplementary Figure 1. A, Comparison of extracellular DNA levels (ng/μl) in untreated and cell‐free (centrifuged) SF samples in 12 patients with inflammatory arthritis. B, Neutrophils from healthy donors (n = 6) were either frozen and thawed to induce necrosis, or stimulated with PMA to induce NETosis. Release of DNA is shown in B and release of PAD activity in C. Statistical analysis was performed using Wilcoxon matched‐pairs signed rank test with horizontal bars representing median values (n = 6; n.s. = not significant; * indicates P < 0.05). D, PAD activity correlated significantly with neutrophil cell counts in the synovial fluid of RA patients (n = 13). Data were analyzed using Spearman's test for correlation (rho = 0.8, P = 0.002). E, PAD activity correlated significantly with the total cell count in untreated synovial fluid of RA patients (n = 15). Data were analyzed using Spearman's test for correlation (rho = 0.72, P = 0.002). [file ART-67-3135-s001.docx]

**SPENGLER**

**Supplementary Methods**

Pellets from the DNase I–treated (D) and stimulated neutrophil (SN) supernatant fractions isolated from 7 patients were cleaned using acetone precipitation and resuspended in 2% SDS. The protein concentrations were determined by the Pierce BCA Protein Assay Kit (Thermo Fisher, USA). 10 µg of each sample were reduced, alkylated and digested in-solution according to Ytterberg et al. 2006 [1]. After zip tipping (Merck Millipore Ltd, Ireland), 1 µg of each sample was separated using C18 RP columns coupled on-line to an LC-MS/MS. The chromatographic separation was achieved using an ACN/water solvent system containing 0.2 % formic acid. The gradient was set up as following: 3−35 % ACN in 89 min, 35−95 % ACN for 5 minutes and 95 % ACN for 8 minutes, all at a flow rate of 300 nl/minute. The samples from 4 patients were analyzed by LTQ Orbitrap Velos ETD and 3 by Q Exactive MS (Thermo Fisher Scientific, Germany). The spectra were acquired on the Velos with a resolution of 60,000 in MS mode, and the top 5 precursors were selected for fragmentation using CID. The spectra on the Q Exactive were acquired with a resolution of 70,000 in MS mode, and the top 10 precursors were selected for HCD fragmentation with a resolution of 17,000.

The data acquired using the Velos and the Q Exactive were quantified in separate analyses. Mass lists were extracted from the raw data using Raw2MGF v2.1.3 and combined into one file using Cluster MGF v2.1.1, programs part of the Quanti work flow [2]. The data was searched against a concatenated version of the human complete proteome database (2013/4) using the Mascot search engine v2.4.1 ([www.matrixscience.com](http://www.matrixscience.com), Matrix Science Ltd., London, UK). The following parameters were used: tryptic digestion (max 2 miscleavages); carbamidomethylation (C) as fixed modification; oxidation (M), pyroglutamate (Q) as variable modifications; 5 ppm as precursor tolerance; 0.25 Da (Velos) or 0.1 Da (Q Exactive) as fragment tolerance. The threshold for 2% FDR was calculated to a peptide score of 22.80 (Velos) and 13.97 (Q Exactive). The quantification was done using the Quanti work flow, which is a quantification software based on extracted ion chromatograms [2]. In short, after searching the combined mgf against the human complete proteome, the resulting dat file and the 6 respectively 8 raw files (representing D and SN fractions from 3 respectively 4 patients) were uploaded into Quanti v2.5.4.3. The following parameters were used: score threshold 20.8 (Velos) or 13.97 (Q Exactive); mass tolerance 10 ppm; minimum peptides/protein 2; maximum allowed deviation in retention time 3% or 5 min; rt order 50 (Velos) or 70 (Q Exactive); only “charge deconvolution” and “use best mascot peptide” were used. The quantitative values were further processed by multiplying the values with the reference abundance and normalizing each sample to the median of the summed intensities for all the samples. The values were finally log 10 transformed. *P* values were calculated using Student’s *t*-test and expectation values were calculated by multiplying the *P* values by the number of observations. Prior to normalization, the serum albumin and keratins were removed.

In order to evaluate the degree of citrullination, the individual analyses were also searched including citrullination (R) and deamidation (N/Q) as variable modifications. Spectra identifying citrullinated peptides were validated manually, by verifying that the precursor mass was correctly assigned and that the modified site was consistent with observed mass shifts in the fragment ions.

**References**

1 Ytterberg AJ, Peltier J-B, van Wijk KJ. Protein profiling of plastoglobules in chloroplasts and chromoplasts. A surprising site for differential accumulation of metabolic enzymes. *Plant Physiol* 2006;**140**:984–97. doi:10.1104/pp.105.076083

2 Lyutvinskiy Y, Yang H, Rutishauser D, *et al.* In silico instrumental response correction improves precision of label-free proteomics and accuracy of proteomics-based predictive models. *Mol Cell Proteomics* 2013;**12**:2324–31. doi:10.1074/mcp.O112.023804

**Supplementary Table 1.** Quantitative comparison of protein levels by mass spectrometry in supernatants of stimulated neutrophils (SN) and DNase I–treated NET fraction of stimulated neutrophils (+DNase-I and/or D) after in vitro induced NETosis.

Supernatant (SN) and DNase I–treated NETs (D) from stimulated neutrophils from 7 donors were digested, analyzed by mass spectrometry (4 donors by LTQ OrbitrapVelos ETD and 3 donors by Q Exactive), searched against the human complete proteome database, and quantified by the Quanti software. The list shows the 286 proteins that could be quantified in both fractions of all patients. The data from the two instruments have been kept separate. The “PROTEIN ID” lists the uniprot accession numbers, “PROTEIN IDs” lists all the accessions sharing peptides with the quantified accession, “log2 (D/SN)” lists the log2 of the ratio of medians of the two fractions, the “P (D vs SN)” lists the p-value using t-test, the “E” show the expectation value (n x p) together with the number of quantified proteins in the dataset. The Bonferroni corrected thresholds for significance are *P* = 1.70E-4 (Velos) and *P* = 9.01E-5 (Q Exactive) respectively.

**Supplementary Table 2.** Citrullinated proteins detected by mass spectrometry in supernatants of stimulated neutrophils (SN) and NET fraction (D) after in vitro induced NETosis

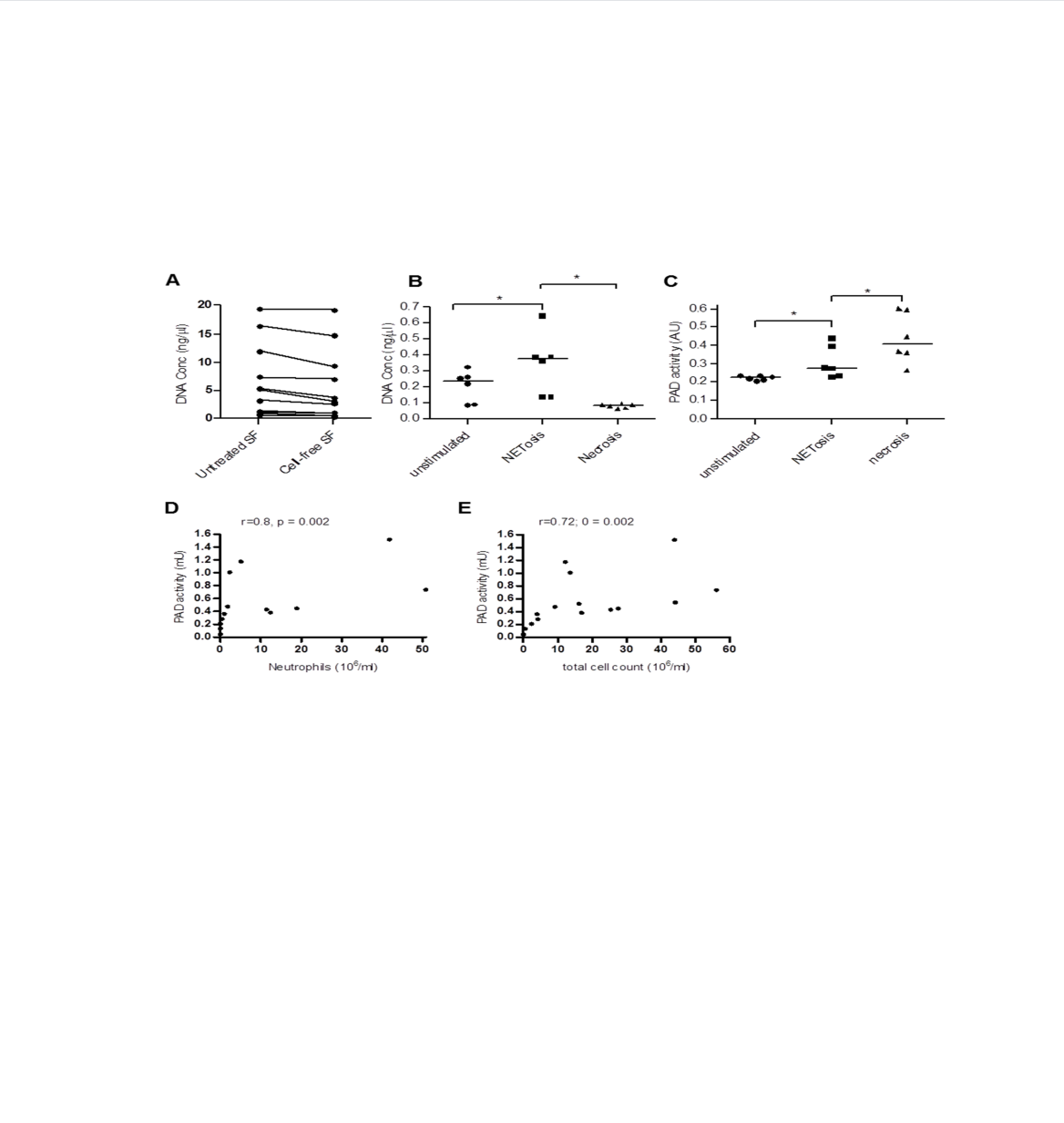


**Supplementary Figure 1. A,** Comparison of extracellular DNA levels (ng/µl) in untreated and cell-free (centrifuged) SF samples in 12 patients with inflammatory arthritis. **B,** Neutrophils from healthy donors (n = 6) were either frozen and thawed to induce necrosis, or stimulated with PMA to induce NETosis. Release of DNA is shown in **B** and release of PAD activity in **C**. Statistical analysis was performed using Wilcoxon matched-pairs signed rank test with horizontal bars representing median values (n = 6; n.s. = not significant; * indicates *P* < 0.05). **D,** PAD activity correlated significantly with neutrophil cell counts in the synovial fluid of RA patients (n = 13). Data were analyzed using Spearman’s test for correlation (rho = 0.8, *P* = 0.002). **E,** PAD activity correlated significantly with the total cell count in untreated synovial fluid of RA patients (n = 15). Data were analyzed using Spearman’s test for correlation (rho = 0.72, *P* = 0.002).
